# Response of the neurovascular unit to brain metastatic breast cancer cells

**DOI:** 10.1186/s40478-019-0788-1

**Published:** 2019-08-19

**Authors:** János Haskó, Csilla Fazakas, Kinga Molnár, Ádám Mészáros, Roland Patai, Gábor Szabó, Ferenc Erdélyi, Ádám Nyúl-Tóth, Fanni Győri, Mihály Kozma, Attila E. Farkas, István A. Krizbai, Imola Wilhelm

**Affiliations:** 1 Institute of Biophysics, Biological Research Centre, Hungarian Academy of Sciences, Temesvári krt. 62, Szeged, 6726 Hungary; 2 Theoretical Medicine Doctoral School, University of Szeged, Szeged, Hungary; 3 Doctoral School of Biology, University of Szeged, Szeged, Hungary; 4 Institute of Experimental Medicine, Hungarian Academy of Sciences, Budapest, Hungary; 5 Department of Physiology, Anatomy and Neuroscience, Faculty of Science and Informatics, University of Szeged, Szeged, Hungary; 6 Institute of Life Sciences, Vasile Goldiş Western University of Arad, Arad, Romania

**Keywords:** Apoptotic and non-apoptotic blebbing, Astrocyte end-foot, Brain metastasis, Cerebral endothelial cell, Endothelial plug, Neurovascular unit, Transcellular pathway, Transendothelial migration, Triple negative breast cancer

## Abstract

Therapeutic resistance of cerebral secondary tumours largely depends on unique aspects linked to the neurovascular unit, especially cerebral endothelial cells and astrocytes. By using advanced microscopy techniques, here we explored novel mechanisms related to the neurovascular unit during extravasation and proliferation of triple negative breast cancer cells in the brain. Metastatic mammary carcinoma cells arrested and elongated within one hour in cerebral microvessels, but their number decreased by almost 80% in the first two days. Interestingly, malignant cells induced vasoconstriction and development of intraluminal endothelial plugs, which isolated invading cells from the circulation. During diapedesis – which usually took place on day four and five after inoculation of the tumour cells – continuity of cerebral endothelial tight junctions remained intact, indicating migration of cancer cells through the transcellular pathway. In addition, metastatic cells induced formation of multiluminal vessels and claudin-5-positive endothelial blebs. However, even severe endothelial blebbing could be reversed and the vessel morphology was restored shortly after the tumour cells completed transendothelial migration. Similar to neuro-inflammatory leukocytes, tumour cells migrated not only through the endothelial layer, but through the glia limitans perivascularis as well. Nevertheless, along with the growth of metastatic lesions by co-option of pre-existing capillaries, astrocytes and astrocyte end-feet were gradually expelled from the vessels to the border of the tumour. Taken together, we identified previously unknown mechanisms involved in the reaction of brain resident cells to invading breast cancer cells. Our results contribute to a better understanding of the complex cross-talk between tumour cells and host cells in the brain, which is essential for the identification of new therapeutic targets in this devastating disease.

**Electronic supplementary material:**

The online version of this article (10.1186/s40478-019-0788-1) contains supplementary material, which is available to authorized users.

## Introduction

Brain metastases, most often originating from lung cancer, breast cancer and melanoma, have a particularly dismal prognosis. Despite significant therapeutic advances in extracranial malignancies, management of brain metastases is still an unmet clinical challenge. Several mechanisms may be responsible for the ineffective treatment strategies, including exclusion of systemic agents by the blood-brain barrier (BBB) [17], dense vascularization of the brain [35] and protective effects of astrocytes on tumour cells [42]. Nevertheless, cells of the neurovascular unit (NVU) have a decisive role in the fate of brain metastatic tumour cells [40].

The concept of the NVU emerged to emphasize the unique intimate relationship between brain cells and the cerebral vasculature [12]. Regulated by input from pericytes, glial cells and neurons, cerebral endothelial cells (CECs) take the centre stage of the NVU to form and maintain the BBB. In contrast to peripheral endothelial cells, CECs are connected to each other by continuous tight junctions (TJs), formed by transmembrane proteins like claudin-5 or occludin and a cytoplasmic plaque. Brain metastatic cells have to take up the challenge of opening or overcoming this tight endothelial barrier before diapedesis through brain microvessels. Accordingly, tumour cells spend several days arrested in the lumen of cerebral capillaries before migrating through the vessel wall [19], despite the severe stress they are exposed to in the blood stream [32]. It is largely unknown what changes are induced in the endothelium during this long time spent by the tumour cells intravascularly. Using in vitro and ex vivo methods, we have previously shown that metastatic cells may up-regulate expression of N-cadherin and mesenchymal markers in CECs during a process called endothelial-mesenchymal transition (EndMT), which can facilitate transmigration of the tumour cells [16]. EndMT is indeed a long-lasting process, which was seen after 2 days in CECs exposed to factors secreted by melanoma or breast cancer cells. Ex vivo, we detected endothelial N-cadherin upregulation in the vicinity of extravasating breast cancer cells and close to the growing metastatic lesions at later time points; however, breast cancer cells migrated through the cerebral endothelium in an N-cadherin-independent manner [10].

Diapedesis itself has been even less characterized in in vivo*/*ex vivo models. It was suggested to take place exclusively from capillaries [19] with the tumour cell being pinched at the transmigration hole on the vessel wall [15]. CECs were shown to extend protrusions covering extravasating mammary carcinoma cells [10], indicating active involvement of the endothelium. However, several questions remain to be answered. What are the changes induced in the cerebral endothelium by tumour cell extravasation in vivo? Is tumour cell-induced endothelial dysfunction reversible or irreversible? Is there any endothelial apoptosis, proliferation or any type of new vessel formation taking place during initial steps of brain metastasis formation?

After completing transvascular migration, metastatic cells can only survive in the brain environment if attached to the abluminal surface of the vessel wall [3]. By this time, tumour cells come in contact with other cells of the NVU. Immediate and persistent peritumoural astrogliosis and microglial reactions are the most important in this respect [40]. It is not known, however, how the glia limitans perivascularis is affected in initial and later phases of brain metastasis formation. Vascular changes during metastatic outgrowth – except for vessel co-option – also need better understanding.

By using real-time in vivo and ex vivo microscopy, here we aimed at unravelling morphological and functional changes in cerebral vessels and cells of the NVU before, during and after transmigration of breast cancer cells through the BBB.

## Material and methods

### Cell culture and in vitro models

4T1 mouse triple negative mammary carcinoma cells were kept in Roswell Park Memorial Institute (RPMI) 1640 medium (Pan Biotech, Aidenbach, Germany) supplemented with 5% foetal bovine serum (FBS, PAA Laboratories, Linz, Austria) and Glutamax (Thermo Fischer Scientific, Waltham, MA, USA). 4T1 cells were transfected with pcDNA3.1(+)/Luc2 = tdT plasmid using Lipofectamine 2000 (Thermo Fischer Scientific) and underwent single-cell cloning, after sorting of red fluorescent cells using a BD FACSAria Fusion flow cytometer (BD Biosciences, San Jose, CA, USA). The media of tdTomato-4T1 cells contained 500 μg/ml G418 (Thermo Fischer Scientific) for further selection and maintenance of red fluorescence. Emerald GFP-expressing EmGFP-4T1 cells were obtained by retroviral transfection and selected on blasticidin S (Sigma-Aldrich, St. Louis, MO, USA). All cell lines were regularly tested for mycoplasma contamination using the MycoAlert Mycoplasma Detection Kit (Lonza, Basel, Switzerland). Only mycoplasma-negative cultures were used for experiments.

Venus-YFP-expressing primary mouse brain endothelial cells (MBECs) were isolated from 6- to 8-week-old FVB/Ant:TgCAG-yfp_sb #27 female mice (obtained from Institute of Experimental Medicine, Budapest, Hungary). After collection of the brains, the meninges were removed and cerebral cortices were cut into small pieces and digested in two steps with collagenase and collagenase/dispase. Microvessel fragments were collected after 10 min 1000 · *g* centrifugation on Percoll (Sigma-Aldrich) gradient, and plated onto fibronectin/collagen-coated dishes. Endothelial cells growing out of the microvessels were cultured in DMEM/F12 (Thermo Fisher Scientific), 10% plasma-derived serum (PDS, First Link, Birmingham, UK) and growth factors. In the first two days, 4 μg/ml puromycin (Sigma-Aldrich) was added to remove contaminating cells.

YFP-MBECs and tdTomato-4T1 cells were used for endothelial-tumour cell co-cultures. First, we cultured endothelial cells on the abluminal side of the filter inserts (Corning-Costar Transwell Clear, Corning, NY, USA, #3450) coated with collagen. Tumour cells were seeded on the luminal side in a number of 4.5 · 10^4^/cm^2^ and co-cultured for 48 h.

### Experimental animals and surgeries

All surgeries were carried out on 8-week old female BALB/c (The Jackson Laboratory) or FVB/Ant:TgCAG-yfp_sb #27 mice. Before every procedure, mice were anaesthetized via inhaled isoflurane 4% (v/v) in oxygen for induction and 1–2% (v/v) for maintenance, from a precision vaporizer (Open Circuit Isoflurane Tabletop System, Stoelting, Dublin, Ireland). Depth of anaesthesia was monitored by toe pinch tests.

For all intravital experiments, cranial windows were used to obtain optical access to the cortex. Briefly, anaesthetized animals were mounted on a stereotaxic frame incorporating a heating pad. Craniotomy (d = 3.5 mm) was performed over the right parietal cortex with a micro drill (H.MH-170, High Speed Rotary Handpiece, Foredom, Blackstone Industries, Bethel, CT, USA) fitted with a 0.5 mm burr, followed by the removal of the dura. In some experiments, astrocytes were labelled by topical application of 10 μM SR101 (Sigma-Aldrich) in Ringer-HEPES solution for 2–3 min before the window installation. A coverslip of 5 mm diameter was then placed over the exposed brain and the edge of the glass was sealed with cyanoacrylate glue. An aluminium plate was glued onto the skull for head fixation. The exposed bone and the aluminium bar were covered with cyanoacrylate glue and dental cement (Unifast III, GC Europe, Leuven, Belgium) to increase stability. A recovery period of at least one month was allowed between implantation of the cranial window and intravital microscopy observation of endothelial-tumour cell interactions. Astrocyte-tumour cell interactions were investigated in a time frame of 30 min to 2 h after cranial window installation due to the temporary astrocyte staining. After recovery, either 10^6^ tdTomato-4T1 cells were inoculated into the right common carotid artery or 3 · 10^6^ tdTomato-4T1 cells were injected intracardially into FVB/Ant:TgCAG-yfp_sb #27 female mice with chronic cranial window for two-photon microscopy, or without craniotomy for ex vivo investigations. BALB/c female mice were intracardially injected with 3 · 10^6^ EmGFP-4T1, then 1 day, 5 days or 10 days later cranial window was installed right before two-photon microscopy imaging.

For ex vivo observations, surgically untouched FVB/Ant:TgCAG-yfp_sb #27 mice received 3 · 10^6^ tdTomato-4T1-cells intracardially. Certain animals were subjected to in vivo proliferation assay and treated intraperitoneally with 5-ethynyl-2′-deoxyuridine (EdU, Thermo Fisher Scientific, 100 mg/kg), a thymidine analogue, 24 h before tissue collection. After 1, 3, 5, 8 or 10 days, animals were anaesthetized and transcardially perfused with phosphate buffered saline (PBS, 10 mM, pH = 7.4), then with Karnovsky’s fixative (for electron microscopy) or 3% paraformaldehyde (for immunofluorescence) in PBS. Brains were removed and post-fixed by immersion in the same fixative at 4 °C overnight. The following day, the fixative was replaced with PBS (for vibratome section) or 30% sucrose solution in 0.1 M phosphate buffer (PB, for frozen sections), and the brains were stored at 4 °C until further processing.

### Intravital two-photon imaging

Mice were anaesthetized with isoflurane and kept on a heating system-incorporated stereotaxic stage. The head was immobilized and positioned via the attached aluminium bar. This stable positioning and the unique pattern of the superficial pial vasculature allowed us to image the same cortex volume over days. Intravital microscopy was carried out with a FEMTO2DAlba microscope (Femtonics, Budapest, Hungary) using a 20x or 60x large working distance water immersion objective using MES software (v4.6.2336, Femtonics). Two-photon excitation was performed using a Mai Tai HP Ti-sapphire laser (Spectra-Physics, San Jose, CA, USA) at 810 nm, which was found optimal for EmGFP and CellTracker Red CMTPX excitation and also adequate for SR101 and at 900 nm, which optimally excited both tdTomato and Venus-YFP. Laser power was set to 10–40% depending on the depth of imaging (0–400 μm from the brain surface). Emission wavelengths were collected by GaAsP photomultipliers. Larger volumes (x: 500 μm; y: 500 μm; z: 250 μm) were recorded with 3 μm vertical steps to evaluate the cell number changes and dynamics of tumour cell intravascular location in the first 48 h following inoculation. To acquire sufficient red fluorescence signal, tdTomato-4T1 cells were also stained with CellTracker Red CMTPX (Thermo Fisher Scientific) for this experimental setup. ImageJ’s “3D Object Counter” plugin was used to assess the changes [1]. High magnification z-stack images (x: 120 μm; y: 120 μm; z: 120 μm, with 1 μm steps) were recorded for studying cell morphology changes and transmigration and no additional labelling of 4T1-tdTomato was applied. Image stacks were auto-levelled, merged and converted to RGB colour in Fiji [28].

### Immunofluorescence and fluorescence microscopy

After fixation, the whole brain was mounted for freezing microtome (Reichert-Jung, Leica Biosystems, Wetzlar, Germany) or vibratome (Leica Biosystems) sectioning and sliced coronally. 50 μm brain sections were collected and stored in PBS with 0.05% sodium azide. Antigen retrieval was either omitted or performed by incubating slices at 85 °C for 60 min in PBS. Permeabilization was performed with 0.5% TritonX-100 in PBS for 30 min at room temperature, followed by blocking with 3% BSA (bovine serum albumin) in PBS. Primary antibody solutions were prepared in 3% BSA and 0.5% TritonX-100-containing PBS. Sections were incubated overnight under slow nutation. The following antibodies were used on vibratome sections: anti-AQP4 (1:100, Santa Cruz Biotechnology, Santa Cruz, CA, USA, #sc-20,812), anti-claudin-5 (1:100, Thermo Fisher Scientific, #35–2500), anti-cleaved caspase-3 (1:50, Cell Signaling, Boston, MA, USA, #9661,), anti-collagen IV (1:100, Abcam, Cambridge, UK, #ab6586) and anti-PECAM-1 (1:120, Novus Biologicals, Centennial, CO, USA, #NB100–2284); on frozen sections: anti-fibronectin (1:100, Abcam, #ab2413), anti-GFAP (1:100, Abcam, #ab7260) and anti-Iba-1 (1:100, Abcam, #ab5076). Sections were extensively washed in PBS, and the secondary antibody solution was afterward applied on them for 60 min at room temperature in the dark. Alexa Fluor 488, 594, 647 anti-rabbit, anti-mouse and anti-goat IgG (Jackson ImmunoResearch, Cambridgeshire, UK and Thermo Fisher Scientific) and STAR RED anti-mouse IgG (Abberior, Göttingen, Germany) were used as secondary antibodies in a dilution of 1:300–1:600 in 3% BSA-containing PBS. Sections were then washed with PBS, counterstained with a colour compatible nuclear staining (Hoechst 33342, Sigma-Aldrich) for 5 min, washed again with PBS, rinsed in water and mounted with an aqueous fluorescent mounting solution, FluoroMount-G media (SouthernBiotech, Birmingham, AL, USA). Visualization of nuclei with DNA synthesis was performed on sections from EdU-treated animals using Click-iT Plus EdU Alexa Fluor 647 Imaging Kit (Thermo Fischer Scientific) following the manufacturer’s instructions. ImageJ’s “3D Object Counter” plugin was used for manual assessment of EdU-positive tumour cells.

Immunohistochemistry and immunofluorescence were visualized with Leica SP5 and Leica SP8 confocal laser scanning microscopes with 63x and 100x oil immersion objectives or a STED (stimulated emission depletion) super-resolution-capable STEDYCON (Abberior Instruments, Göttingen, Germany) built on an Axio Observer Z1 inverted epifluorescence microscope (Zeiss, Oberkochen, Germany) equipped with an alpha Plan-Apochromat 100x/1.46 oil immersion objective.

### Preparation of ultrathin sections and transmission electron microscopy (TEM)

TdTomato-4T1-bearing FVB/Ant:TgCAG-yfp_sb #27 mouse brains were sectioned using a VT1000S (Leica Biosystems) vibratome. 100 μm sections were collected and post-fixed for 5 h in Karnovsky’s fixative at room temperature. After post-fixation, sections were cut into 1–2 mm^2^ pieces. Specimens containing tdTomato-4T1 cells were selected for further processing. Samples were rinsed and post-fixed in 2% OsO_4_. After dehydration with a graded series of ethanol, the samples were embedded in epoxy resin (Durcupan ACM, Sigma-Aldrich) and polymerized at 55 °C for 48 h. Ultrathin sections (50 nm) were prepared with an Ultracut UCT (Leica Biosystems) and contrasted with 2% uranyl acetate (Electron Microscopy Sciences, Hatfield, PA, USA) and 2% lead citrate (Electron Microscopy Sciences), then analysed with a JEM-1400Flash transmission electron microscope (JEOL, Tokyo, Japan) fitted with an 8 MP Matataki Flash CCD camera (JEOL).

### Western-blot

4T1-tdTomato cells and YFP-Venus MBECs were co-cultured for 48 h on the two sides of the filter inserts and lysed separately in radioimmunoprecipitation assay buffer. After 30 min incubation on ice, cell lysates were centrifuged at 13,000 · *g* for 15 min at 4 °C. Protein concentration was determined with bicinchoninic acid (BCA) (Santa Cruz Biotechnology). Laemmli buffer was added to the samples, followed by heating at 95 °C for 3 min. Prepared samples were electrophoresed using standard denaturing SDS-PAGE procedures and blotted on polyvinylidene difluoride (0.2 μm pore size from Bio-Rad, Hercules, CA, USA and the 0.45 μm pore size from BioTrace, Pall Corporations, Port Washington, NY, USA) membranes (β-actin and fibronectin, respectively). Afterwards, the non-specific binding capacity of the membranes was blocked with 3% BSA or 5% non-fat milk in TBS-T (Tris-buffered saline with 0.1% Tween-20). Membranes were incubated with primary antibodies diluted in TBS-T: anti-β-actin (1:1000, Santa Cruz Biotechnology, #sc-47,778) or fibronectin (1:1000, Abcam, #ab2413). Blots were washed in TBS-T three times for 10 min. Horseradish peroxidase (HRP)-conjugated secondary antibodies were diluted in TBS-T as follows: 1:3000 anti-rabbit IgG and anti-mouse IgG (Jackson ImmunoResearch) and added for 1 h and then washed again in TBS-T. Immunoreaction was visualized with Clarity Chemiluminescent Substrate (Bio-Rad, Hercules, CA, USA) in a ChemiDoc MP System (Bio-Rad). Densitometry analysis was performed with the Image Lab Software, version 5.2 (Bio-Rad).

## Results

### Metastatic breast cancer cells arrest and elongate rapidly in cerebral microvessels

**Fig. 1:**
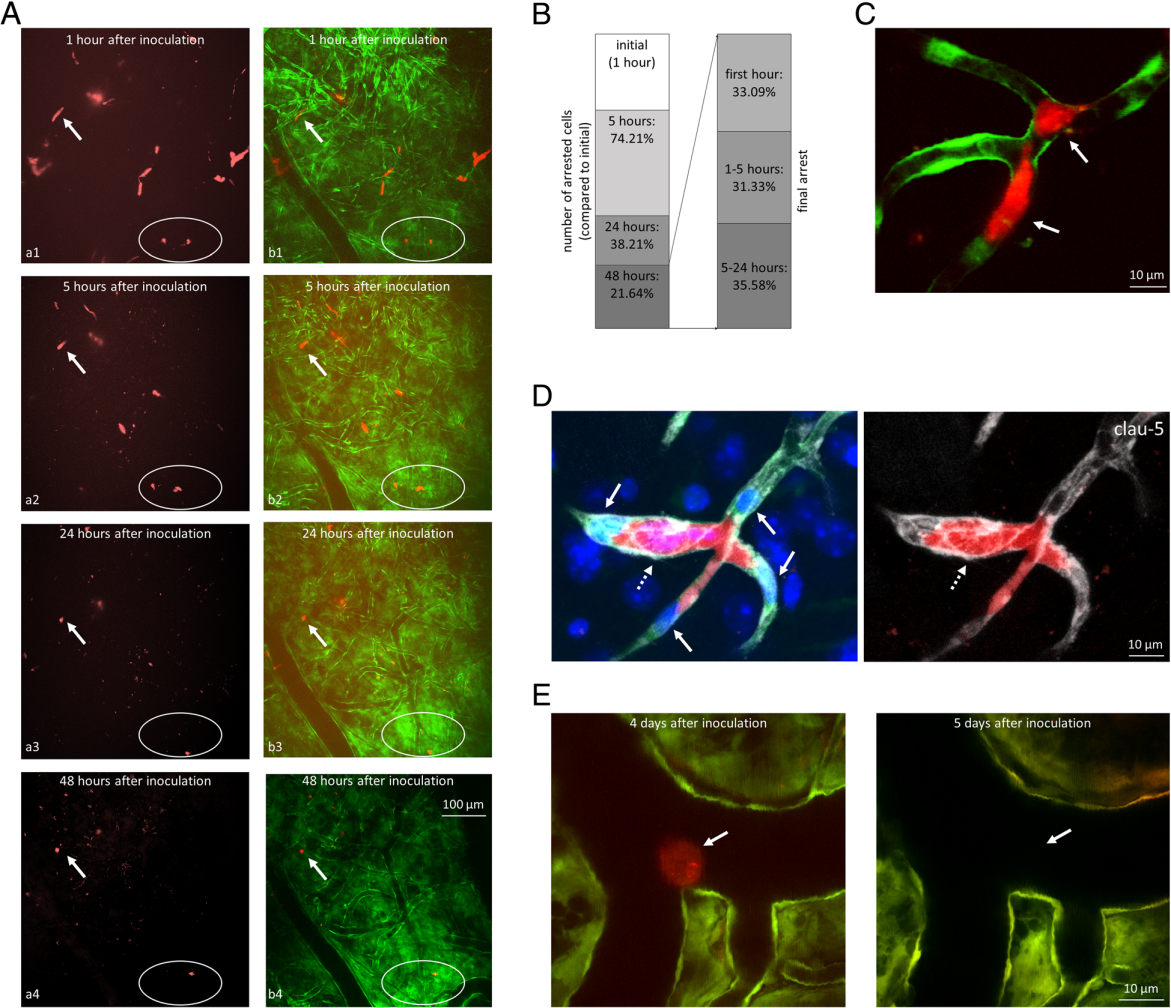
Arrest of breast cancer cells in the cerebral vasculature. **a**: Representative two-photon microscopy z-projection images of tdTomato-4T1 cells arrested in the cerebral microvasculature of FVB/Ant:TgCAG-yfp mice. The same region of the same mouse was imaged at all time points. **a1-a4**: tdTomato-4T1 cells; **b1-b4**: merged red (tdTomato-4T1) and green (Venus-YFP-labelled endothelial cells) channels. Arrow indicates the same cell at all time points. Encircled region shows cells changing position. **b**: Quantitative representation of breast cancer cells arrested in the brain 1 h, 5, 24 and 48 h after inoculation (first panel). Percentage of surviving cells finding the final position in the vascular lumen in the first hour, between 1 and 5 h and between 5 and 24 h (second panel). *N* = 3 animals, *n* = 3 brain regions (500 · 500 · 250 μm^3^ x · y · z) of each animal. **c**: Representative two-photon micrograph (z-projection) of tdTomato-4T1 cells arrested at a vascular branching point in the parietal cortex (5 h after inoculation). Arrows indicate elongated tumour cells. **d**: Claudin-5 immunostaining (grey) at 4 days after the injection of tdTomato-4T1 cells into the circulation of mice having Venus-YFP-labelled endothelial cells; confocal z-projection. Arrows indicate endothelial nuclei protruded into the lumen; dashed arrow shows dilated capillary. **e**: Tumour cell arrested in a pial artery in two-photon micrographs (z-projections) captured at 4 and 5 days after inoculation, respectively. Red = tumour cells (tdTomato), green = endothelium (YFP), blue = nuclei (Hoechst)

As a unique feature of brain metastasis formation [40], tumour cells spend several days in cerebral capillaries before proceeding to diapedesis through the vessel wall [19]. During this phase [15], metastatic cells may change their initial resting position in the vascular lumen. By monitoring the same brain regions in living mice using intravital two-photon microscopy, we explored the number of tumour cells/cell clusters disappearing, changing position and remaining in resting phase in the first 48 h after inoculation of triple negative, tdTomato-expressing 4T1 breast cancer cells. The relatively large volume scanned with the consequently lower magnification, and the lack of nuclear staining did not allow for clear detection of morphology changes and determination of the number of tumour cells in an intravascular cell cluster. However, we could still assess that metastatic cells gained an elongated morphology already within 1 h after the injection of tumour cells into the arterial circulation (Fig. 1a). Some cells remained in the same position for two days, while others changed their initial location and disappeared from or appeared in the scanned volumes (Fig. 1a and b). The number of initially arrested tumour cells/cell clusters (imaged at 1 h after tumour cell inoculation) decreased to 74.21 ± 21.95% at 5 h, to 38.21 ± 31.60% at 24 h and to 21.64 ± 5.59% at 48 h (Fig. 1b), as detected by monitoring the same three regions in the parietal cortex of three mice each by two-photon microscopy. We next wanted to assess the time of final arrest of the tumour cells, i.e. when they reached their resting position, from where they did not move inside the capillary lumen anymore. We found that 33.09 ± 26.97% of the cells adhered in the first hour, 31.33 ± 30.23% arrested between 1 h and 5 h and the remaining 35.58 ± 28.18% of the cells attached to the luminal side of the endothelium between 5 h and 24 h, and remained in the same location by 48 h post-inoculation (Fig. 1b). The surviving cells did not change their position after 24 h. These numbers indicate that the firm cell arrest happens in the first 24 h and, most probably, the majority of the cells find their long-term intravascular location in the initial hours.

Tumour cells arrested preferentially in vascular branching points (Fig. 1c) and left the junctions intact in the first 4 days, as assessed by PECAM-1 (platelet and endothelial cell adhesion molecule) (Additional file 1: Figure S1) and claudin-5 immunostainig (Fig. 1d) and TEM (Additional file 1: Figure S2). Breast cancer cells have been previously found to arrest exclusively in microvessels (capillaries and post-capillary venules) of the brain [19]. Outside the brain parenchyma, we could find breast cancer cells stuck in subarachnoid large vessels; however, these cells never elongated and disappeared after a few days without extravasation (Fig. 1e).

### Arrested metastatic cells induce vasoconstriction, endothelial plug formation and obstruction of cerebral microvessels

**Fig. 2:**
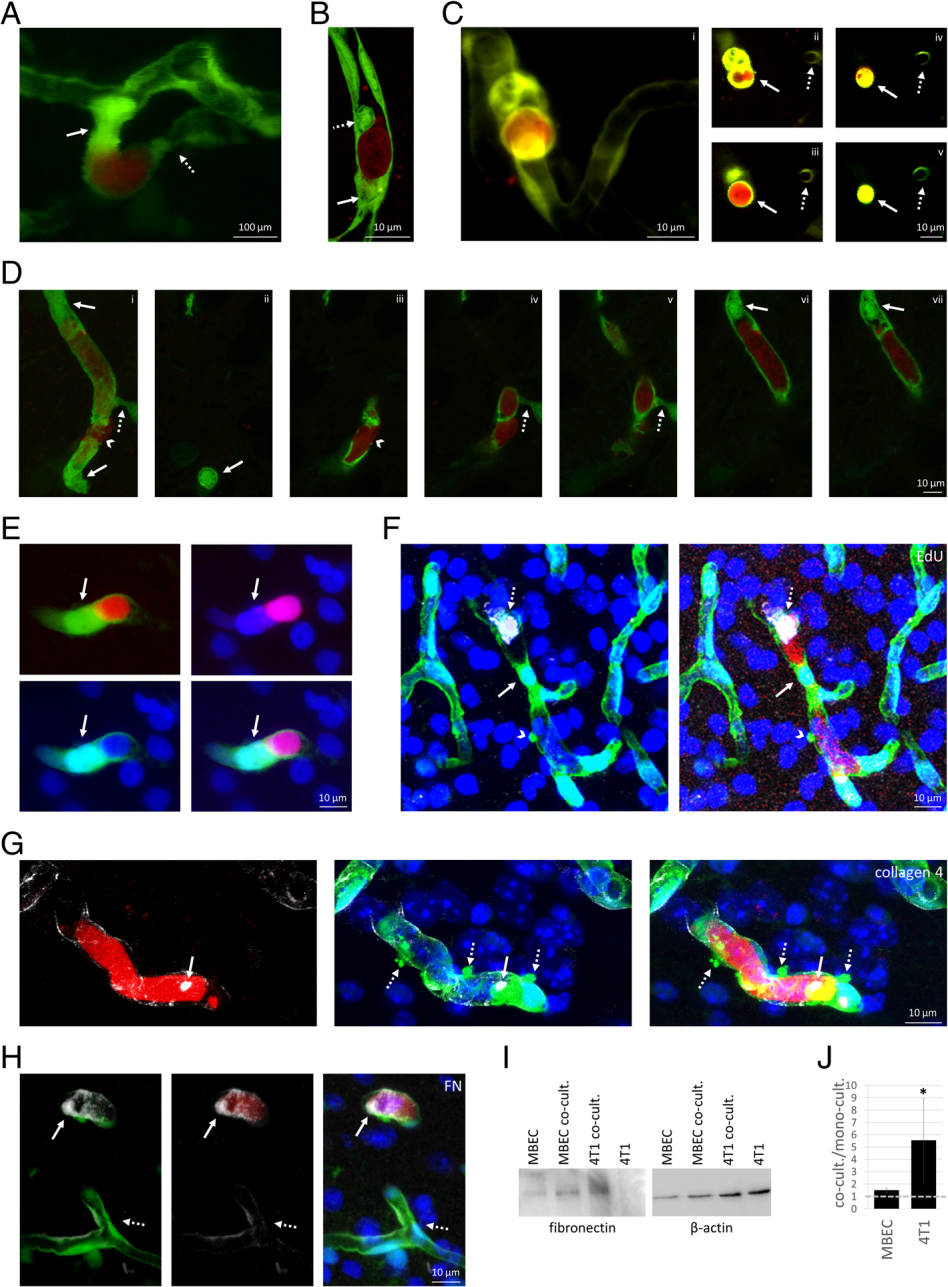
Vascular changes induced by metastatic cells before extravasation into the brain parenchyma. **a**: Two-photon micrograph (z-projection) of an endothelial plug (arrow) and vascular constriction (dotted arrow) in the neighbourhood of an arrested breast cancer cell, on day 5 after tumour cell injection. **b**: Confocal z-projection of endothelial plugs completely (arrow) or partially (dotted arrow) obstructing the cerebral capillary up- and downstream of an arrested metastatic cell (day 4 after inoculation). **c**: Complete obstruction of the vessel lumen by the tumour cell-induced endothelial plug in 3D (**i**) and cross-section (**ii-v**) two-photon microscopy images (day 5). Arrow indicates tumour cell-induced plug formation in different optical slices. Dotted arrow points to a capillary not affected by cancer cells. **d**: 3D representation (**i**) and z-sections (**ii-vii**) of endothelial plugs (arrows) and vasoconstriction (dotted arrow) closing all capillary branches in the proximity of a transmigrating tumour cell (arrowhead) (confocal micrograph, day 5). **e**: Confocal z-projection indicating the endothelial nucleus in the plug (arrow) obstructing the capillary next to the cancer cell. **f**: EdU-positivity (grey) of intraluminal tumour cell, but not of endothelial nucleus in the plug (confocal z-projection). **g**: Up-regulation of collagen secretion (arrow, grey) in the neighbourhood of arrested carcinoma cells (confocal z-projection). Dashed arrows indicate endothelial blebs. **h**: Up-regulation of fibronectin secretion (FN, grey) in the neighbourhood of arrested carcinoma cells (confocal z-projection, arrows). Dashed arrows indicate basal fibronectin expression in non-affected capillaries. **i**: Representative western-blot image of fibronectin expression in mouse brain endothelial (MBEC) and tdTomato-4T1 cells in mono- and co-culture. B-actin was used as loading control. **j**: Fibronectin protein levels normalized to β-actin in co-cultured vs. mono-cultured MBECs and tdTomato-4T1 cells (average ± SD) (western-blot quantification). *N* = 3 independent experiments. * *P* < 0.05 (*t*-test). Red = tumour cells (tdTomato), green = endothelium (YFP), blue = nuclei (Hoechst)

After 24–48 h spent in the lumen of parenchymal vessels, mammary carcinoma cells induced vasoconstriction and formation of endothelial plugs, which protruded into the lumen both upstream and downstream of the cancer cells (Fig. 2a). High-resolution confocal microscopy images indicated both partial and complete obstructions of the vascular lumen in the neighbourhood of the tumour cells (Fig. 2b). We could clearly see complete vessel obstruction by the tumour cell-induced plug in living animals as well, in a series of two-photon microscopy optical cross-sections (Fig. 2c). When tumour cells arrested at vascular branching points, vessels in all directions were obstructed by plugs and constrictions (Figs. 1d and 2d).

By examining 6–9 cancer cells/animal in the brains of 10 tumour-inoculated mice using confocal microscopy, we assessed the prevalence of vascular obstructions in the neighbourhood of the cancer cells on day 4 after inoculation. Majority of the tumour cell-containing microvessels were completely (61.03%) or partially (23.90%) closed by endothelial plugs. In addition, 9.01% of them were obstructed by vasoconstriction and only 6.06% remained open. These numbers indicate that it is a general phenomenon in brain capillaries that tumour cells are isolated by endothelial cells from the circulation.

Plugs of protruded endothelial bodies contained the nuclei of the endothelial cells (Fig. 2e). We hypothesized that these plugs were formed by endothelial division; however, endothelial nuclei in the plugs were EdU negative (Fig. 2f). As a positive control, we detected EdU-positive endothelial cells not involved in plug formation and not coming in contact with carcinoma cells (Additional file 1: Figure S3). Therefore, tumour cell-associated plugs were formed as the result of endothelial reorganization and not cell division. In addition, overexpression of abnormally localized claudin-5 and PECAM-1 was observed in plug-forming endothelial cells (Additional file 1: Figure S4 and S5), suggesting junctional reorganization and possibly compromised endothelial polarity. Interestingly, a large part of the intraluminal tumour cells was EdU positive (Fig. 2f) at each time point assessed: 62.50% at 24 h, 51.85% at 3 days and 40.54% at 5 days after tumour cell injection. Despite the high prevalence of EdU-positive intraluminal tumour cells, no increase in tumour cell number was observed from 24 h to days 3 or 5. Supposedly, some of the intravascular tumour cells became polyploid/multinucleated, which could help their survival and metastasis formation [22]. These large cells formed in the capillary lumen after day 3, dilated the vessel, as shown in Fig. 1d.

In the vicinity of the plugs and intravascular tumour cells, accumulation of matrix proteins was observed in the lumen, like type 4 collagen (Fig. 2g) and fibronectin (Fig. 2h). Besides the possible contribution of circulating matrix proteins [38], fibronectin was also overexpressed in the tumour cells (Fig. 2i and j). Tumour cells expressed even more fibronectin after completing the diapedesis (Additional file 1: Figure S6).

### Extravasation occurs either via small pores or through large openings on the vessel wall, through the transcellular pathway, often from multiluminal vessels

After 3–8 days spent attached to the luminal surface of the cerebral endothelium, typically on day 4 or 5, metastatic cells contracted and migrated through the vessel wall into the brain parenchyma. Interestingly, we observed a few transmigrating tumour cells that were proliferating/EdU positive (Additional file 1: Figure S7).

**Fig. 3:**
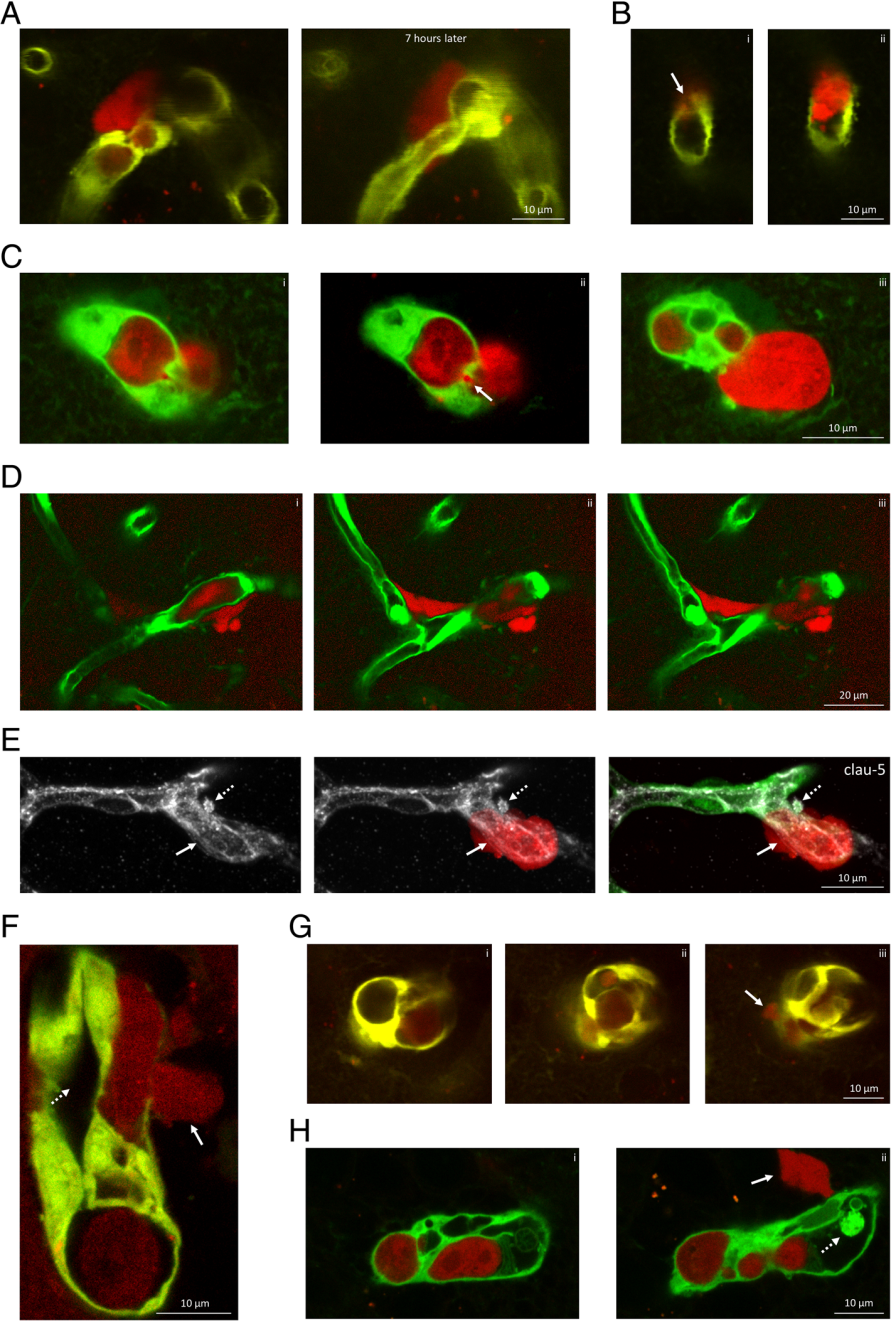
Extravasation of triple negative mammary carcinoma cells into the brain. **a**: Transmigration of a tdTomato-4T1 cell through a small pore in a constricted capillary on day 4 after inoculation of the tumour cells. Representative z-projection of a two-photon micrograph. 7 h later, the extravasation is completed and the tumour cell is attached to the extraluminal surface of the vessel. **b**: Cross-section representation of the transendothelial migration of a breast cancer cell through a small pore (arrow) on day 4 after inoculation of the tumour cells. The shown two-photon microscopy images (**i**, **ii**) are optical sections of the same tumour cell. **c**: Confocal z-sections (**i**-**iii**) of a tumour cell migrating out of a multiluminal vessel on day 4 after inoculation of the tumour cells. Arrow indicates transmigration pore observed on a single optical section (**ii**). **d**: Transmigration through a large opening on the vessel wall, as represented in two-photon microscopy z-sections (**i**-**iii**). **e**: Claudin-5 staining (grey) of intact TJs in the neighbourhood of a tumour cell (arrow) extravasating from a cerebral capillary (confocal z-projection). Dashed arrow indicates a claudin-5-positive bleb on the basolateral side of the endothelium. **f**: Representative two-photon micrograph of a tumour cell (arrow) migrating through the wall of a multiluminal vessel, represented in longitudinal section. Dashed arrow indicates the collateral lumen. **g**: Two-photon microscopy cross-sections (**i**-**iii**) of a capillary having multiple lumens. Arrow indicates extravasating tumour cell. **h**: Confocal microscopy cross-sections (**i**-**ii**) of a capillary having multiple lumens. Arrow indicates extravasating tumour cell. Dashed arrow shows an endothelial plug. Red = tumour cells (tdTomato), green = endothelium (YFP)

In contrast to the long time spent intravascularly, the diapedesis itself was relatively rapid, completed in a few hours and was accompanied by resolution of the obstructions (Fig. 3a). Our two-photon and confocal microscopy investigations revealed two types of transvascular migration. Metastatic breast cancer cells either diapedesed through small pores (Fig. 3a, b and c) or large discontinuities opened on the vessel wall (of 10 μm diameter in average) (Figs. 2d and 3d). By observing 14 transmigrations in the brains of living animals using two-photon microscopy, we counted 10 diapedesis events which occurred through pores < 5 μm (71.43%) and 4 through large fenestrations (28.57%).

Using in vitro models, we have previously shown that breast cancer cells are able to migrate transcellularly, leaving the TJs intact [10]. Here we explored this possibility in our mouse model, and found that continuity of TJs, represented by claudin-5 staining, remained intact in the vicinity of transmigrating cells (Fig. 3e). This suggests that breast cancer cells are able to take the transcellular route of transmigration through the BBB in vivo.

Another phenomenon that we observed was that cancer cells induced formation of additional vascular channels (Fig. 3f-h). Multiluminal vessels containing several channels or chambers (ranging from a few up to 15) were observed in 5 of 14 (35.71%) transmigrations tracked in two-photon microscopy. Smaller transmigration pores were formed on the thinned wall of one of the tubes of the multichannelled endothelium. Besides splitting of the capillary, rarely, we could see the growth of a collateral bypassing the tumour cell-bearing vessel (Additional file 1: Figure S8).

### Diapedesis of cancer cells is accompanied by blebbing of both tumour and endothelial cells

**Fig. 4:**
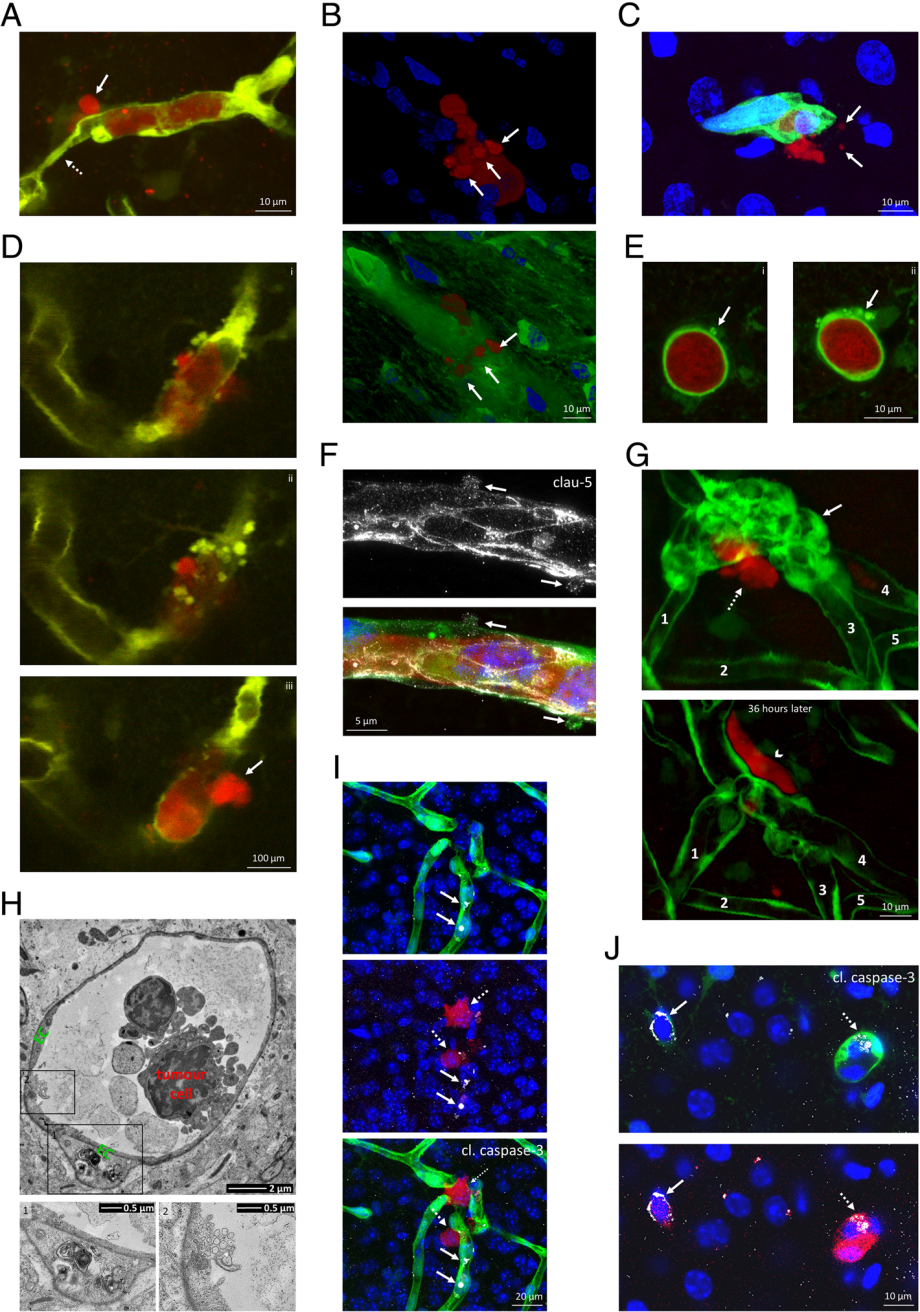
Blebbing of metastatic and endothelial cells of cerebral vessels. **a**: Representative two-photon micrograph (z-projection) of a blebbing breast cancer cell (arrow) migrating from the lumen of the capillary to the brain parenchyma. Dotted arrow indicates vasoconstriction. **b**: 3D confocal image representation of a blebbing metastatic cell projecting out the blebs through the vessel wall during initial phase of transendothelial migration. **c**: Confocal microscopy z-projection indicating blebs detaching from the tumour cell (red) resulting in large oncosome-like vesicles (arrows). **d**: Endothelial apoptotic blebbing during diapedesis of a metastatic cell (arrow) through the capillary wall, as represented in two-photon microscopy z-sections (**i**-**iii**). **e**: Basolateral blebs (arrows) in endothelial cells of a cerebral capillary hosting a breast cancer cell, as represented in confocal microscopy z-sections (**i**-**ii**). **f**: Claudin-5-positivity (grey) of endothelial blebs (arrows). STED (claudin-5) and confocal (blue, green and red channels) z-projection images are shown. **g**: Two-photon micrograph (z-projection) presenting intensive blebbing of cerebral microvessels during transmigration of cancer cells. Arrow indicates blebbing, dashed arrow shows extravasating tumour cell. 36 h later, the tumour cell reached an extravascular position (arrowhead), while intensive blebbing is almost completely resolved. Numbers indicate matching vessels in the two images. **h**: Transmission electron micrograph of a cerebral capillary and an intravascular tumour cell. Insets show mitochondrial damage (**1**) and blebbing (**2**) of the endothelium. EC = endothelial cell. **i**: Vesicle-like cleaved caspase-3 positivity (grey) in endothelial cells close to metastatic cells, as shown in a confocal z-projection. Arrows indicate cleaved caspase-3-positive endothelial cells, dashed arrows mark tumour cells. **j**: Cleaved caspase-3 positivity (grey) of endothelial cells and metastatic cells, as shown in a confocal z-stack. Arrow indicates cleaved caspase-3 positivity in endothelial cells, dashed arrows mark cleaved caspase-3-positive tumour cells. Red = tumour cells (tdTomato), green = endothelium (YFP), blue = nuclei (Hoechst)

Migration of the tumour cells through the cerebral endothelium was frequently accompanied by blebbing of metastatic and/or endothelial cells (Fig. 4a-h). Intensive blebbing of intravascular tumour cells (Fig. 4a-c) was frequent, observed in 11 of 14 transmigrations (78.57%) imaged in two-photon microscopy. During extravasation, cancer cells usually protruded their blebbing membrane part through the transmigration pore (Fig. 4a and b), while the nucleus was left behind in the lumen and got through the vessel wall in the terminal stage of the diapedesis. Blebbing tumour cells often released extracellular vesicles (Fig. 4c), resembling large oncosomes [21].

In the neighbourhood of extravasating cancer cells, endothelial blebbing (Figs. 2g, 3e and 4d-h) was similar in frequency to tumour cell blebbing, present in 11 of 14 events observed by two-photon microscopy (78.57%) and in 10 of the 11 cases of tumour blebbing (90.91% overlap). Ranging from a few extraluminal blebs (Figs. 2g, 3e, 4e and f) to moderate or intensive vacuolization (Fig. 4d, g and h), blebbing of CECs was always linked to the presence of the tumour cells. Endothelial blebs were claudin-5 positive, while the endothelium still showed a continuous claudin-5 staining marking the intact TJs (Fig. 4f). Even severe endothelial blebbing could be reversed and the vessels were restored in 1–2 days after the tumour cells completed transendothelial migration (Fig. 4g).

Besides the non-apoptotic blebbing of the cerebral endothelium described above, which is most probably an important element of the active involvement of CECs in metastatic extravasation, signs of apoptotic blebbing and endothelial cell death could also be detected. On the one hand, we observed degenerated mitochondria in blebbing endothelial cells (Fig. 4h). On the other hand, we saw cleaved caspase-3-positive endothelial cells in the neighbourhood of carcinoma cells (Fig. 4i and j). However, this was a relatively rare event, observed in 5 of 21 cases in day 4. Endothelial active caspase-3 staining usually appeared in vesicle-like structures, showing the typical morphology of cell death. We assume that endothelial cell death might provide passage for the tumour cells through the formation of large openings on the vessel wall. The majority of tumour cells were usually cleaved caspase-3 negative (Fig. 4i); however, in 5 of 21 cases, we detected apoptotic tumour cells as well in day 4 (Fig. 4j).

### Tumour cells migrate through the glia limitans perivascularis, but exclude astrocytes from the growing metastatic lesion

**Fig. 5:**
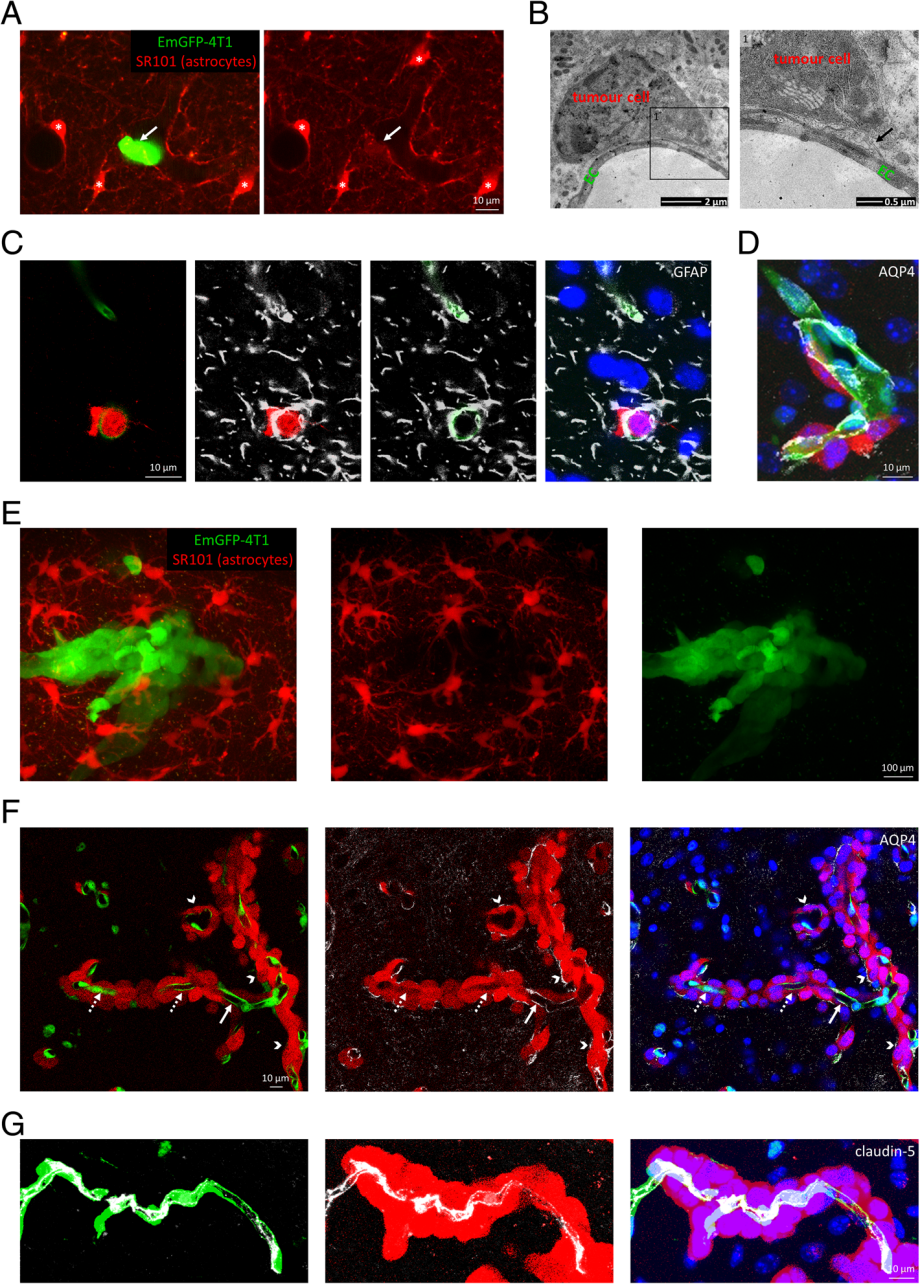
Interaction of metastatic cells with astrocytes and proliferation in the brain parenchyma. **a**: Two-photon microscopy z-projections indicating a metastatic breast cancer cell (green) breaking the glia limitans perivascularis. SR101-positive astrocytes are marked in red; asterisks indicate cell bodies of astrocytes. Capillaries are delineated by end-feet of astrocytes; arrow indicates disruption of the continuity in the glia limitans during extravasation of the tumour cell. **b**: Transmission electron micrograph showing an extravasated tumour cell attached to the extraluminal surface of a cerebral capillary (day 5). Inset (**1**) highlights astrocyte end-foot (arrow) between the tumour cell and the capillary endothelium. **c**: Representative confocal z-projections showing GFAP-positive astrocyte end-feet covering capillaries in initial phases of diapedesis of metastatic cells. Red = tumour cells (tdTomato), green = endothelium (YFP), blue = nuclei (Hoechst), grey = GFAP. **d**: Confocal z-projection showing AQP4-positive astrocyte end-feet co-opted by extravasated metastatic cells. Merged image of blue (nuclei), green (endothelium), red (tumour cells) and grey (AQP4) channels. **e**: Astrocytes expelled from the growing metastatic lesion on day 10 after inoculation, as shown in a two-photon z-projection image. Red = astrocytes (SR101), green = tumour cells (EmGFP). **f**: AQP4-positive astrocyte end-feet gradually retracted from the capillary wall to the surface of the metastatic tumour. Confocal z-projection of metastatic lesions from the brain of an animal on day 10. Red = tumour cells (tdTomato), green = endothelium (YFP), blue = nuclei (Hoechst), grey = AQP4. Arrows show vascular AQP4 staining, dotted arrows indicate absence of vascular AQP4 staining, while arrowheads point to AQP4 staining on the surface of the tumour. **g**: Intact TJs in vessels co-opted by growing metastatic tumours on day 10 after, as shown in a confocal z-projection image. Red = tumour cells (tdTomato), green = endothelium (YFP), blue = nuclei (Hoechst), grey = claudin-5

As a next step, interactions of tumour cells with astrocytes and especially astrocyte end-feet were investigated during and after the extravasation. We observed that the extravasated part of breast cancer cells passed the glia limitans perivascularis right after breaching the endothelial wall, as our in vivo (Fig. 5a) and ex vivo (Fig. 5b-d) observations indicate. During extravasation, carcinoma cells opened the glia limitans formed by an almost continuous layer of astrocyte end-feet covering the extraluminal surface of the vessels (Fig. 5a). Tumour cells reaching an extraluminal position remained attached to the vessel wall, incorporating astrocyte end-feet, as shown in TEM (Fig. 5b) and confocal microscopy images (Fig. 5c and d). Both the astrocyte-specific glial fibrillary acidic protein (GFAP) and the end-foot marker aquaporin-4 (AQP4) were localized between the partly or completely extravasated tumour cell and the capillary endothelium.

After migrating through the capillary wall, tumour cells started to divide by co-opting one or two capillaries, which developed an abnormal, disorganized, bent morphology (Additional file 1: Figure S9). Tumour cells proliferated rapidly; however, endothelial proliferation was almost never observed (Additional file 1: Figure S10), suggesting that blood supply of the tumours was primarily provided by vascular co-option and not neo-angiogenesis. As metastatic lesions were growing, astrocytes were expelled from the tumour mass (Fig. 5e and Additional file 1: Figure S11). In parallel, reactive astrocytes surrounding metastatic lesions gradually retracted their end-feet from the vessels to the parenchymal border of the tumour, which gained a discontinuous end-foot coverage (Fig. 5f). It should be noted that the tumour-covered endothelium still showed a continuous claudin-5 staining, indicating the presence of complete TJs in the absence of the perivascular astrocyte sheath (Fig. 5g). We have also observed activated microglia [30] surrounding transmigrating and extravasated, but not intravascular cancer cells (Additional file 1: Figure S12).

## Discussion

The microenvironment is increasingly recognized as a profound determinant of tumour progression and therapeutic outcome. Composed of neurons, glial and vascular cells and protected by the BBB, the brain environment is very special, generally able to host only a few cancer types. Among these, triple negative breast cancer is one of the most relevant, having the worst outcome among mammary carcinoma subtypes [13].

Due to the complex anatomy and intercellular communication network, in vitro models are less suitable to study mechanisms related to the brain environment. In addition, early steps of brain metastasis development cannot be observed in human, because of the detection limit of current diagnostic tools. Xenograft models are devoid of a full immune response, while injection of tumour cells into the brain parenchyma is not recapitulating the extravasation step [34]. Therefore, we applied an in vivo animal model and followed the interactions of brain resident cells with metastatic breast carcinoma cells. Since only a limited number of metastatic lesions were observed in the peripheral organs of the mice, our in vivo setup is a relevant model of brain metastasis formation with the condition that tumour heterogeneity and interspecies differences (translation from mouse to human) are not addressed.

Among cells of the NVU, CECs and astrocytes are the most active in immediately responding to and continuously associating with invading tumour cells [15, 19]. Both CECs and astrocytes have a Janus-faced attitude, bearing both tumour-destructive and -protective mechanisms [40]. Much less is known about the role of pericytes, which regulate the permeability of the blood-tumour barrier [20] and contribute to connective tissue accumulation in the metastases [33].

During the long-lasting arrest in the lumens of cerebral capillaries, several changes may take place both in cancer and in endothelial cells. EndMT is one mechanism, the development of which is a slow process, lasting for a few days [16]. EndMT is characterized by loss of TJ proteins, switch from VE- to N-cadherin and up-regulation of mesenchymal markers, like collagen, fibronectin and α-smooth muscle actin. We have previously described up-regulation of N-cadherin in CECs of tumour cell-hosting capillaries; however, N-cadherin proved to be dispensable for the extravasation of breast cancer cells [10]. Here we observed no reduction in the expression of claudin-5 – the protein forming the backbone of endothelial TJs – throughout the metastatic process. Although we saw up-regulation of extracellular matrix proteins, which are also markers of EndMT; however, mainly in tumour and not in endothelial cells. Therefore, we conclude that EndMT is probably not a mandatory mechanism involved in extravasation of triple negative mammary carcinoma cells to the brain, at least in mouse.

Although the extravasation occurred late (after 4–5 days or even later), surviving and finally diapedesing cells reached their final position in the capillary lumen early (in the first 24 h). The majority of arrested tumour cells disappeared in the first 2 days. Time-dependent reduction of the number of arrested tumour cells may be a consequence of mechanical stress, anoikis or immune attacks [32]. Mechanical stress may be primarily caused by deformation of the tumour cells in the narrow vessels, because fluid shear is practically completely excluded by the obstruction of the lumen by the tumour cell itself and the endothelial plugs, described here. Flow may not only induce shear stress, but may also influence different steps of tumour cell extravasation. In a zebrafish model, reduced flow was found to promote early arrest of tumour cells, while increased flow enhanced extravasation because integrin-dependent adhesion forces rapidly exceeded shear forces [9]. Therefore, isolation of tumour cells from the blood flow may have complex consequences on the metastatic process.

According to our results, anoikis seems to be a rare event, since we could seldom find cleaved caspase-3-positive tumour cells. Cell death was also rarely observed in endothelial cells coming in contact with cancer cells, leading to the formation of large outlets for extravasation of the tumour cells through the vessel wall. However, the majority of metastatic transmigrations occurred through small (< 5 μm) pores. In more than one third of the cases, the lowest resistance point through which the diapedesis took place was found by the tumour cell after the development of multichannelled capillaries. Using in vitro models, we have previously shown that brain endothelial cells extend filopodia-like membrane protrusions towards breast cancer cells, incorporating them and facilitating the transcellular route of transendothelial migration [10]. Endothelial protrusions covering extravasating cells were seen in vivo as well [10], and this seems to be the mechanism of the formation of multiple channels within a vessel. This type of transmigration has been previously described as “endothelial covering-type extravasation” in a zebrafish model of HeLa tumour formation [14] and “endothelization” in a mouse pulmonary melanoma metastasis model [18, 26].

In fact, the process of endothelial protrusion formation and consequent multilumination of the vessel resembles the first step of intussusceptive angiogenesis [5], when endothelial bridges are formed [25]. Collateral capillaries spanning the tumour cell-affected vessel may form through this mechanism. However, according to our observations, the main tumour vascularization mechanism [41] in mouse brain metastases is vessel co-option. During this process, cancer cells proliferate attached to the abluminal surface of already existing capillaries [15] interacting with the basement membrane [3, 36].

It is still a question of debate whether tumour cell migration through the vessel wall induces a reversible or irreversible damage of the endothelium. Using an in vitro model, we have previously suggested that melanoma cells induce apoptosis of CECs [8]. However, melanoma and breast cancer cells seem to have a distinct behaviour in the brain, showing different transmigration efficiencies and routes (paracellular of melanoma and mainly transcellular of breast cancer) [10, 23]. Here we observed that triple negative breast cancer cells might induce endothelial death in the mouse brain during their extravasation; however, more often, the endothelium could recover even from severe structural changes. These changes included blebbing of the endothelial cell membrane, which was observed with a frequency of almost 80% in the neighbourhood of tumour cells. Blebbing is usually associated with apoptosis [4]; however, non-apoptotic membrane blebbing has also been described in a wide variety of cell types in response to multiple stimuli [7]. Non-apoptotic endothelial blebbing has been observed in oxidative stress [37] and adhesion [24]. The role of endothelial bleb formation in the metastatic process remains to be elucidated. Moreover, it is also not clear whether the extent of blebbing (from a few basolateral vacuoles to complete restructuration of the vessel wall) has any impact on the tumour cells.

Nevertheless, blebbing involves rearrangement of the membrane and the cytoskeleton, a process dependent on the Rho family of small GTPases, especially activation of Rho-kinase (ROCK) [7]. In apoptotic cells, activation of ROCK by caspase-3 seems to be responsible for bleb formation [29]. In addition, ROCK activation has also been linked to tumour cell motility. In line with this, we frequently observed tumour cell blebbing during extravasation in the brain. Generally, the blebbing membrane of the tumour cell was the first to squeeze through the vessel wall, followed by the cytoplasm and the nucleus. The bleb-associated mode of tumour cell motility that does not require proteolysis and is associated with a rounded cell morphology, the so-called amoeboid migration, is dependent on ROCK activation [27]. However, our previous in vitro results indicated that fostering the amoeboid migration by Rac inhibition hampers not only melanoma, but also breast cancer cell transmigration through brain endothelial monolayers [23, 39]. It remains to be established how small GTPase signalling and blebbing in endothelial and tumour cells affect breast cancer brain metastasis formation.

Another interesting observation of our study is that tumour cells breach the glia limitans perivascularis during their extravasation. This is important because immune cells – diapedesis of which is used as a reference in deciphering mechanisms of extravasation of tumour cells [26, 31] – also have to migrate through the glia limitans perivascularis to induce neuro-inflammation [6, 11]. However, as a metastatic lesion is growing, AQP4-positive astrocyte end-feet are disappearing from the vessel to cover the surface of the tumour, from which reactive astrocytes are completely expelled. Since in parallel with vanishing from the capillaries, AQP4 accumulates at the border of the tumour, we conclude that this is rather an extraction of astrocyte end-feet from the vessels than loss of polarization, as previously suggested [20]. Interestingly, claudin-5 staining of the vessels remained continuous even after loss of direct contact with astrocyte end-feet, probably due to the presence of pericytes [2]. Indeed, disappearance of astrocyte foot processes from metastatic vessels did not show any correlation with the permeability [20]. Although we cannot exclude the possibility that some vessels become leaky due to partial opening of the TJs, our results suggest that this is not mandatory in brain metastatic lesions.

## Conclusions

By using advanced microscopy techniques – including monitorization of tumour cell movements in the brains of living animals – we observed novel mechanisms related to cells of the NVU, namely CECs and astrocytes, in brain metastasis formation of triple negative mammary carcinoma. These mechanisms include:

-isolation of the invading cell from the circulation by vessel obstruction and formation of multiluminal vessels;
-tumour cell-induced blebbing of endothelial cells followed by recovery after completion of transmigration;
-transcellular migration of tumour cells through single brain endothelial cells and breaching of the glia limitans perivascularis;
-retraction of astrocyte end-feet from the co-opted vessel to the surface of the tumour with preserved TJ integrity of CECs.

Although it is largely unclear which of these are pro- and which are anti-metastatic, the large number of transmigration-related phenomena may explain the difficulty in finding a therapeutic target in this devastating disease.

## Additional file

Additional file 1: **Figure S1.** Intact PECAM-1 staining in the proximity of arrested metastatic cells. **Figure S2.** Preserved TJs in the proximity of a metastatic cell engaged to transendothelial migration. Arrows indicate intact TJs. Transmission electron micrographs. Images (**1**) and (**2**) are higher magnifications of insets shown in first panel. **Figure S3.** Proliferating endothelial cell in a cerebral capillary distant to tumour cells. Arrow indicates an EdU-positive endothelial cell. **Figure S4. ** Claudin-5 staining of the plug-forming endothelium. **Figure S5.** PECAM-1 staining in CECs involved in plug formation. **Figure S6.** Up-regulated expression of fibronectin (FN) by a tumour cell extravasated into the brain parenchyma. **Figure S7.** Diapedesing EdU-positivetumour cell. Arrow indicates transmigration pore on the capillary wall. **Figure S8.** Collateral capillary (arrow) bridging the vessel damaged by a metastatic cell. Two-photon micrograph (z-stack). **Figure S9.** Tortuous vessels in brain metastatic tumours as shown in two-photon (**1**) and confocal (**2**) z-projections. Co-option of two capillaries is shown in (**1**). **Figure S10.** Proliferating tumour, but not endothelial cells in the metastatic lesion on day 10 after inoculation. **Figure S11.** Exclusion of astrocytes from the growing tumour in the brain. Two-photon microscopy z-section of image presented in Fig. 5f. Merged image of green (tumour, EmGFP) and red (SR101-positive astrocytes) channels. Arrows point to astrocyte end-feet localized outside the tumours, dashed arrow indicates astrocyte end-feet covering intact cerebral capillaries, asterisks are astrocyte bodies, arrowheads show lumens of capillaries co-opted by the tumours. **Figure S12.** Microgliosis around extravasating tumour cells. Arrows show Iba-1-positive microglia surrounding extravasated tumour cells. Dashed arrow indicates absence of microglial reaction around the intravascular tumour cell. **Figures S1, S3-5, S3 (2) and S12:** confocal z-projection images; blue = nuclei (Hoechst staining), green = endothelium (YFP), red = tumour cells (tdTomato), grey = specific stainin. (TIF 49922 kb)

## Data Availability

Not applicable.

## Abbreviations

AQP4: Aquaporin-4
BBB: Blood-brain barrier
BSA: Bovine serum albumin
CEC: Cerebral endothelial cell
EdU: 5-ethynyl-2′-deoxyuridine
EndMT: Endothelial-mesenchymal transition
GFAP: Glial fibrillary acidic protein
NVU: Neurovascular unit
PBS: Phosphate-buffered saline
PECAM-1: Platelet and endothelial cell adhesion molecule (CD31)
ROCK: Rho-kinase
STED: Stimulated emission depletion
TBS-T: Tris-buffered saline with 0.1% Tween-20
TEM: Transmission electron microscopy
TJ: Tight junction
