# Supplementary figures and images for: Response of the neurovascular unit to brain metastatic breast cancer cells
Source: Acta Neuropathol Commun. 2019 Aug 19;7:133. doi: 10.1186/s40478-019-0788-1 (PMC6699134; doi:10.1186/s40478-019-0788-1)

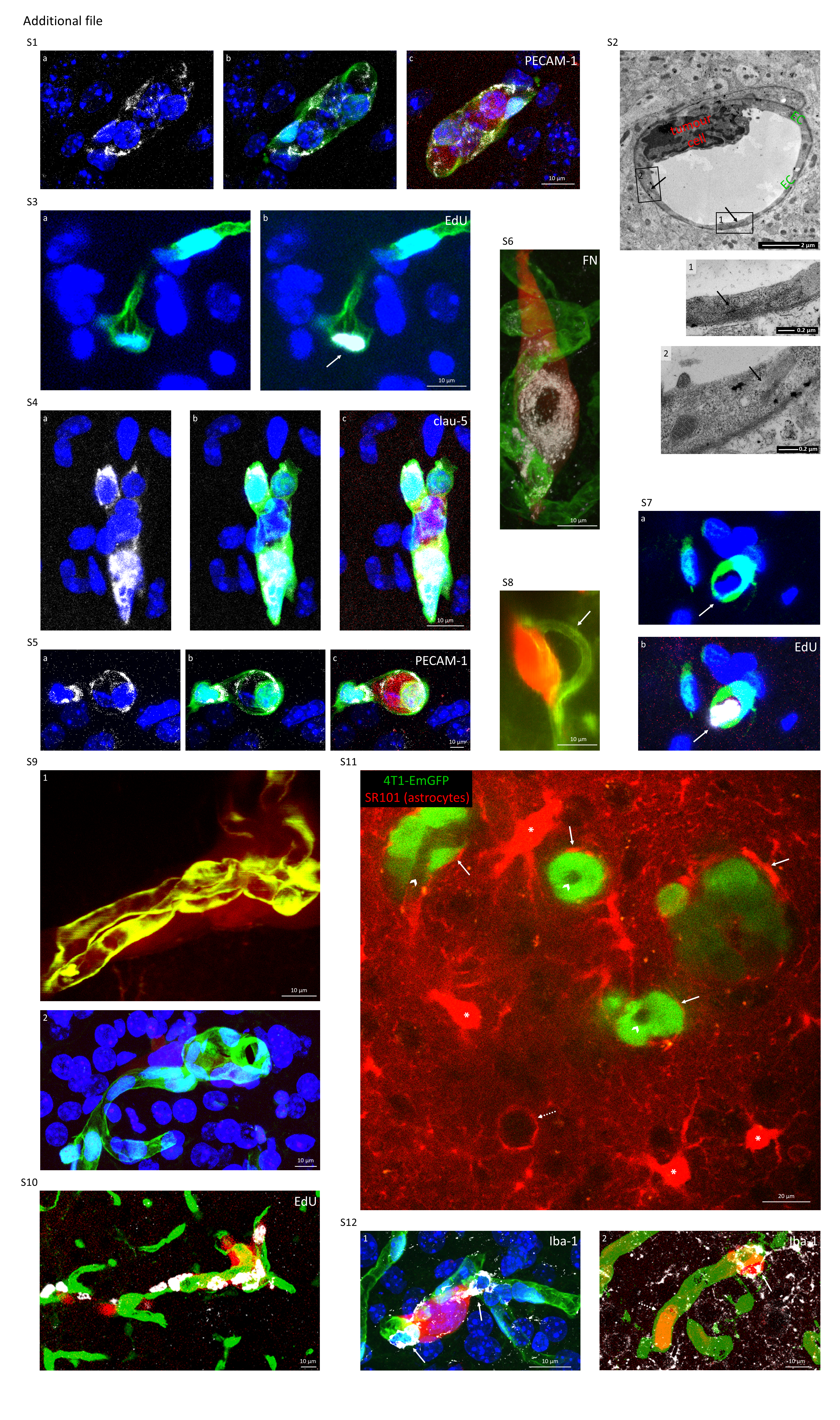

Supplement: Supplementary file 1 — Figure S1. Intact PECAM-1 staining in the proximity of arrested metastatic cells. Figure S2. Preserved TJs in the proximity of a metastatic cell engaged to transendothelial migration. Arrows indicate intact TJs. Transmission electron micrographs. Images (1) and (2) are higher magnifications of insets shown in first panel. Figure S3. Proliferating endothelial cell in a cerebral capillary distant to tumour cells. Arrow indicates an EdU-positive endothelial cell. Figure S4. Claudin-5 staining of the plug-forming endothelium. Figure S5. PECAM-1 staining in CECs involved in plug formation. Figure S6. Up-regulated expression of fibronectin (FN) by a tumour cell extravasated into the brain parenchyma. Figure S7. Diapedesing EdU-positivetumour cell. Arrow indicates transmigration pore on the capillary wall. Figure S8. Collateral capillary (arrow) bridging the vessel damaged by a metastatic cell. Two-photon micrograph (z-stack). Figure S9. Tortuous vessels in brain metastatic tumours as shown in two-photon (1) and confocal (2) z-projections. Co-option of two capillaries is shown in (1). Figure S10. Proliferating tumour, but not endothelial cells in the metastatic lesion on day 10 after inoculation. Figure S11. Exclusion of astrocytes from the growing tumour in the brain. Two-photon microscopy z-section of image presented in Fig. 5f. Merged image of green (tumour, EmGFP) and red (SR101-positive astrocytes) channels. Arrows point to astrocyte end-feet localized outside the tumours, dashed arrow indicates astrocyte end-feet covering intact cerebral capillaries, asterisks are astrocyte bodies, arrowheads show lumens of capillaries co-opted by the tumours. Figure S12. Microgliosis around extravasating tumour cells. Arrows show Iba-1-positive microglia surrounding extravasated tumour cells. Dashed arrow indicates absence of microglial reaction around the intravascular tumour cell. Figures S1, S3-5, S3 (2) and S12: confocal z-projection images; blue = nuclei (Hoechst staining), gre [file 40478_2019_788_MOESM1_ESM.tif]
